# Surgical preferences in anterior cruciate ligament reconstruction: A cross-sectional study in a low-middle income country

**DOI:** 10.1371/journal.pone.0327966

**Published:** 2025-07-28

**Authors:** José Fernando Sánchez-Carbonel, José Calmet-Rojas, Fabriccio J. Visconti-Lopez, Diego Urrunaga-Pastor, David Torres-Manrique

**Affiliations:** 1 Department of Orthopaedic Sports Medicine, Technical University of Munich, Munich, Germany; 2 Department of Orthopedics and Trauma Surgery, Asklepios Klinik Wandsbek, Hamburg, Germany; 3 Universidad Peruana de Ciencias Aplicadas, Lima, Peru; 4 Carrera de Medicina Humana, Facultad de Ciencias de la Salud, Universidad Científica del Sur, Lima, Peru; 5 Clínica Anglo Americana, Lima, Peru; 6 Sociedad Peruana de Ortopedia y Traumatología – SPOT, Lima, Peru; University of South Carolina, UNITED STATES OF AMERICA

## Abstract

**Introduction:**

Anterior cruciate ligament reconstruction (ACLR) is one of the most common surgical procedures in sports orthopedics; however, there is a lack of literature related to this topic in Peru. This study aimed to describe the current surgical preferences in ACLR among Peruvian orthopedic surgeons.

**Methods:**

An analytical cross-sectional study based on an online survey sent to 500 Peruvian orthopedic surgeons, supplemented with physical surveys distributed during the “IX OrthoTrauma Update” congress held in October 2022 in Lima, Peru. The survey collected demographic characteristics (e.g., residence area, health care sector), employment characteristics (e.g., experience as orthopedic surgeon, experience in ACLR), and surgical preferences for ACLR (choice of femoral tunnel drilling technique, choice of graft, tourniquet use). A total of 162 surgeons responded (32.4%).

**Results:**

A total of 134 responses were analyzed. 50% (n = 67; 95% CI: 41.5–58.5) of orthopedic surgeons had more than 10 years of experience, and 53.7% (n = 72; 95% CI: 45.2–62.1) had less than 5 years of experience in ACLR. Anteromedial portal was the most popular choice for femoral tunnel drilling (62.7%; n = 84; 95% CI: 54.1–70.5). Additionally, 85.1% preferred using autograft and 83.6% (n = 114; 95% CI: 77.9–90.2) of surgeons reported a preference to obtain the graft from hamstrings.

**Conclusions:**

To our knowledge, this is the first report to describe the surgical preferences for ACLR among Peruvian orthopedic surgeons, revealing a predominant preference for autografts and hamstring tendon grafts, as well as a high frequency of the anteromedial (AM) portal technique and variability in tourniquet use.

## Introduction

The anterior cruciate ligament (ACL) is the primary stabilizer of the anterior translation of the tibia, and plays a secondary role in controlling knee rotation [1]. ACL injuries are among the most common sports-related injuries. In the United States alone, approximately 120,000 ACL reconstructions are performed annually, reflecting a substantial clinical burden in high-demand populations [2]. Therefore, ACL reconstruction (ACLR) is one of the most common surgical procedures in sports orthopedics, widely performed to address instability and prevent further joint damage [3,4].

The torn ligament is removed and replaced with a graft, which may come from the patient’s own tissue (autograft), from a tissue bank (allograft) or a combination of both (hybrid graft) [5]. The autograft is preferred for its shorter operative time and faster recovery compared to allografts, which are associated with higher failure rates and longer incorporation times [4,6,7]. On the other hand, the success and failure rates of hybrid grafts are similar to those of autografts [8]. Another critical surgical decision involves the femoral tunnel drilling technique, with options including transtibial (TT), anteromedial (AM), outside-in (OI), and all-inside (AI) approaches [7,9]. Studies suggest that the AM technique better replicates the anatomical femoral tunnel compared to TT, while OI and AI techniques offer advantages in specific patient populations, such as those with skeletal immaturity or obesity [9–19].

Studies on new trends in orthopedic surgery are important since they guide orthopedic surgeons in the choice of the most adequate technique and investigate and improve techniques to provide better and more effective treatment [20,21]. Currently, there is literature describing the surgical preferences in ACLR in the United States, Europe and Asia [3,4,22,23]. However, no similar research has been found in Latin America or Peru. Furthermore, the findings of the previously mentioned studies cannot be extrapolated to Peru because the resources available, population type, and level of sports activity are not the same as in high-income countries [24,25]. It is therefore important to investigate the preferences of Peruvian orthopedic surgeons in a context completely different to that of previous studies.

This study describes the surgical preferences of Peruvian orthopedic surgeons regarding ACLR techniques and graft choices. We hypothesize that Peruvian orthopedic surgeons’ ACLR differ from those in high-income countries, potentially influenced by factors such as resource availability, training background, and institutional settings.

## Methods

### Design and study population

**Fig 1:**
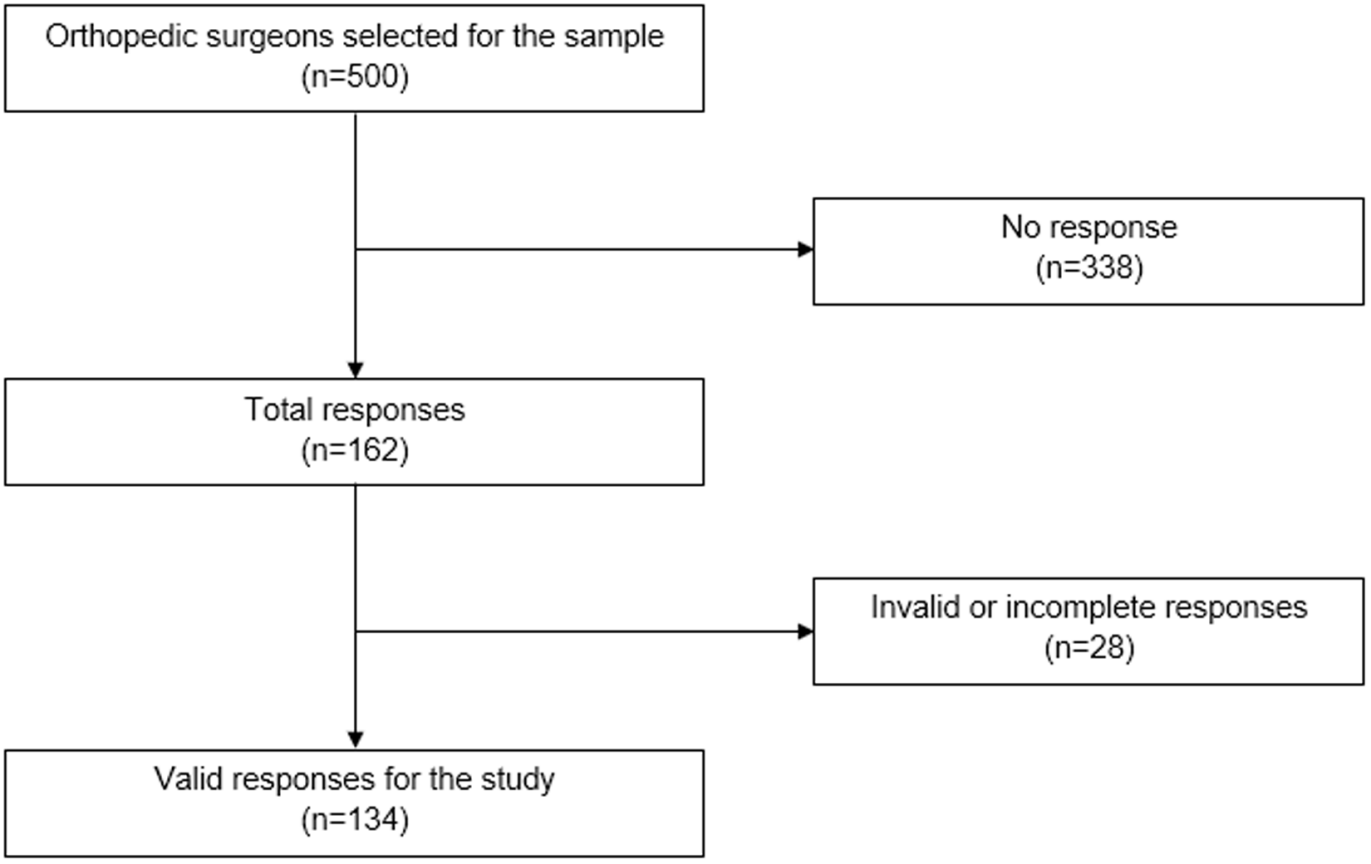
Flowchart of the study participant selection.

We conducted an analytical cross-sectional study based on surveys sent to a self-selected sample of 500 participants between March and October 2022. The survey was sent to orthopedic surgeons who are members of the Peruvian Society of Orthopedics and Traumatology (SPOT) or who have attended a congress organized by this society and have access to email (Fig 1). This was a census-based study aimed at surveying the entire population of orthopedic surgeons affiliated with SPOT. Therefore, no sample size calculation was performed, as the objective was to include the entire available population.

### Variable and measurement

We designed an ad hoc instrument in a web application (REDCap®) based on the experience of two authors, an experienced orthopedic surgeon and a medical resident in orthopedic surgery (DTM & JSC) and a previous study [22]. This instrument was composed of two sections: a general data section and a section related to the preferences of ACLR. The instrument was validated following a Delphi methodology by 6 health professionals, including 3 epidemiologists, and 3 orthopedic surgeons. Similarly, a pilot sample of 5 orthopedic surgeons was included to evaluate the clarity and understandability of the content. The modifications suggested were gradually incorporated until the instrument was approved by consensus by the group of experts.

We divided the questionnaire into two parts: 1) Demographic and work-related variables: sex (male, female), age (under 30, 30–40, 41–50, 51–60, 61–70, and over 70 years), area of residence (Lima, Coast without Lima, Highlands and Jungle) and main health care working sector (public hospital, social insurance hospital, private clinic and armed forces); 2) Surgical preferences for ACLR: years as a trauma specialist (less than five years, between five and ten years, between 11 and 20 years, more than 20 years), years of experience in ACLR (less than five years, between five and ten years, between 11 and 20 years, more than 20 years), the time interval in which ACLR is usually performed from day of injury (less than seven days, seven to 13 days, 14–20 days, 21–28 days and more than 28 days), femoral tunnel drilling arthroscopic technique (AM, TT, modified TT, OI, AI), time interval in which physical therapy is usually indicated after ACLR (the next day, two to seven days after, eight to 14 days after, 15–21 days after, after 21 days), type of graft used most frequently for ACLR (autograft, allograft, hybrid graft), choice of anatomical graft site (hamstrings, patellar tendon, quadriceps tendon), tourniquet use during ACLR (always, sometimes, never) and use of any component after performing ACLR (intra-articular corticosteroid, tranexamic acid, platelet-rich plasma, none, others).

### Data collection and analysis

The data collection was carried out between 21^st^ March and 31^st^ October 2022. The survey was sent to the institutional emails of the SPOT member surgeons during March and April 2022. Additionally, physical surveys were distributed to all the SPOT members at the “IX OrthoTrauma Update” congress held in October 2022 in the city of Lima, Peru. An identifier was considered to exclude duplicate responses. These surveys were subsequently digitized by the authors in REDCap®. The data collected was exported from the REDCap® platform to a spreadsheet in Microsoft Excel 2020. The analyzed database did not contain identifiers. All analyses were performed using Stata version 18.0 (StataCorp, TX, USA). We used descriptive statistics to summarize the data, reporting absolute and relative frequencies, and 95% confidence intervals for all categorical variables.

### Ethical approval

The study protocol was approved by the Ethics Committee of the Universidad Peruana de Ciencias Aplicadas (FCS-SCEI/051-02-22). Respondents to the self-applied survey were provided with detailed information on the conditions, objectives, and benefits of the study. Written informed consent was obtained from all participants prior to their involvement in the study. They were informed that participation was voluntary, and they could withdraw from the study at any time without any consequences. The survey did not contain any data that could personally identify the participants with their responses.

## Results

From the total of 500 orthopedic surgeons members of the SPOT, we obtained 162 responses (32.4%) and excluded 28 (17.3%) due to incomplete responses. We analyzed the responses of 134 orthopedic surgeons (Fig 1). Among the sample, 93.3% (n = 125; 95% CI: 87.3–96.7) were male, 47% (n = 63; 95% CI: 38.7–55.5) were younger than 40, and 27.6% (n = 37) were older than 60 years. Also, 61.2% (n = 82; 95% CI: 52.6–69.1) resided in Lima, followed by 33.6% (n = 45; 95% CI: 26.0–42.1) in the coast. Moreover, 53% (n = 71; 95% CI: 44.5–61.3) worked mainly in the private sector and 42% (n = 57; 95% CI: 34.4–51.1) worked in a public or social insurance hospital. Only 4.5% (n = 6; 95% CI: 2.0–9.7) reported working in the armed forces (Table 1).

**Table 1:** Demographic characteristics and surgical preferences for anterior cruciate ligament reconstruction in the study participants (n = 134).

Variables	N	%	95% CI
***Demographic and work-related variables***			
**Sex**			
Male	125	93.3	87.5-96.5
Female	9	6.7	3.5-12.5
**Age (years)**			
Less than 30	5	3.7	1.5-8.7
30–40	58	43.3	35.1-51.9
41–50	34	25.4	18.7-27.9
51–60	27	20.1	14.1-27.9
61–70	10	7.5	4.0-13.4
More than 70	0	0	–
**Area of residence**			
Lima	82	61.2	52.6-69.1
Coast without Lima	45	33.6	26.0-42.1
Highlands	6	4.5	2.0-9.7
Jungle	1	0.7	0.1-5.2
**Main health care working sector**			
Public Hospital	28	20.9	14.8-28.7
Social Insurance Hospital	29	21.6	15.4-29.5
Private Clinic	71	53	44.5-61.3
Armed Forces	6	4.5	2.0-9.7
***Surgical preferences for anterior cruciate ligament reconstruction***			
**Choice of graft**			
Autograft	114	85.1	77.9-90.2
Allograft	18	13.4	8.6-20.4
Hybrid graft	2	1.5	0.4-5.8
**Choice of anatomical graft site**			
Hamstrings	112	83.6	76.2-89.0
Patellar tendon	22	16.4	11.0-23.8
Quadriceps tendon	0	0	–
**Tourniquet use**			
Always	38	28.4	21.3-36.6
Sometimes	50	37.3	29.5-45.9
Never	46	34.3	26.7-42.8
**Use of some component after performing anterior cruciate ligament reconstruction**			
Intra-articular corticosteroid	17	12.7	8.0-19.5
Tranexamic acid	8	6.0	3.0-11.6
Platelet-rich plasma	8	6.0	3.0-11.6
Hyaluronic acid	10	7.4	4.0-13.4
None	88	65.7	57.2-73.3
Others	3	2.2	0.7-6.8

95% CI: 95% confidence interval.

**Fig 2:**
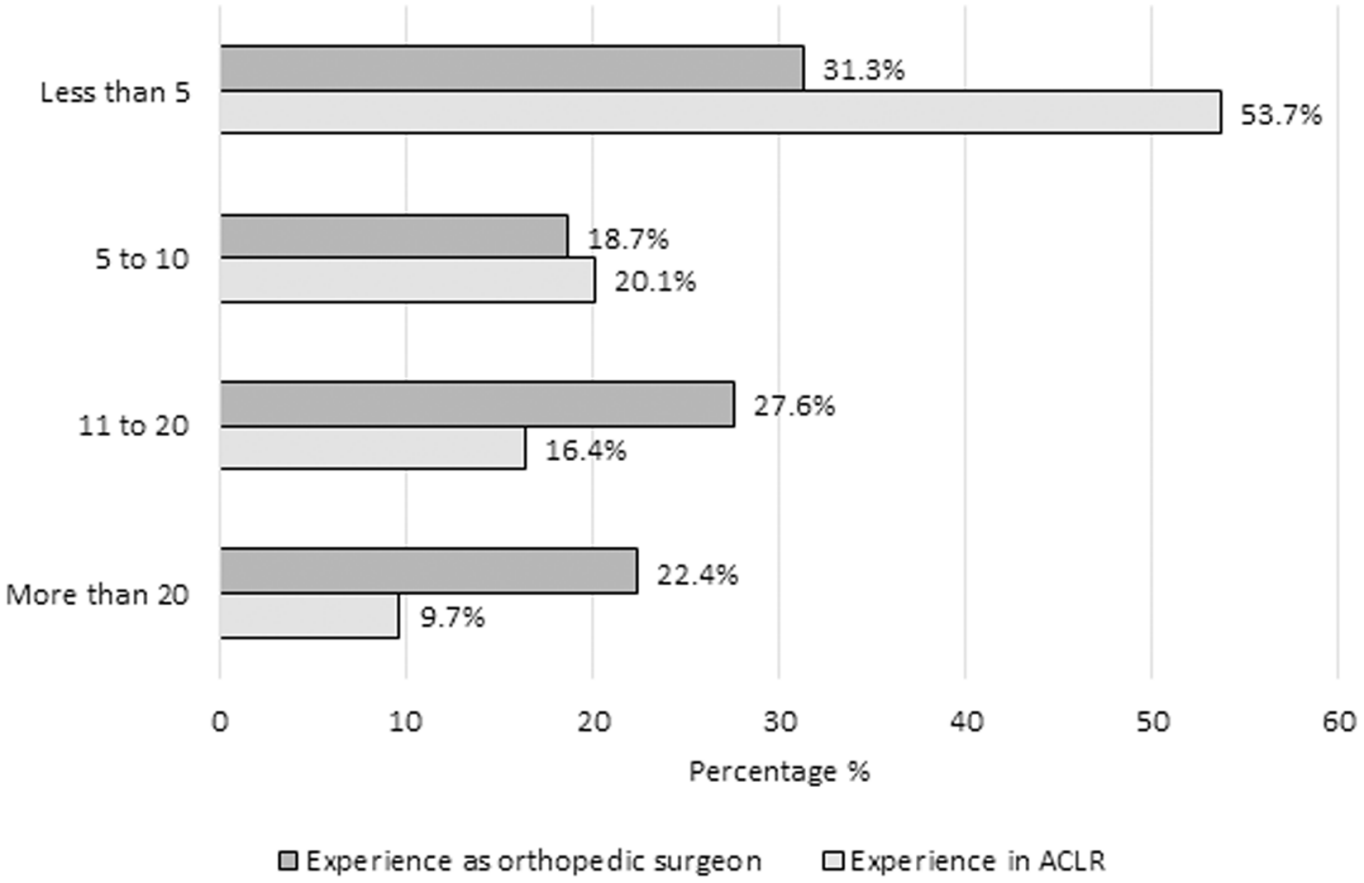
Distribution of years of experience among orthopedic surgeons in general practice and specifically in anterior cruciate ligament reconstruction (n = 134).

**Fig 3:**
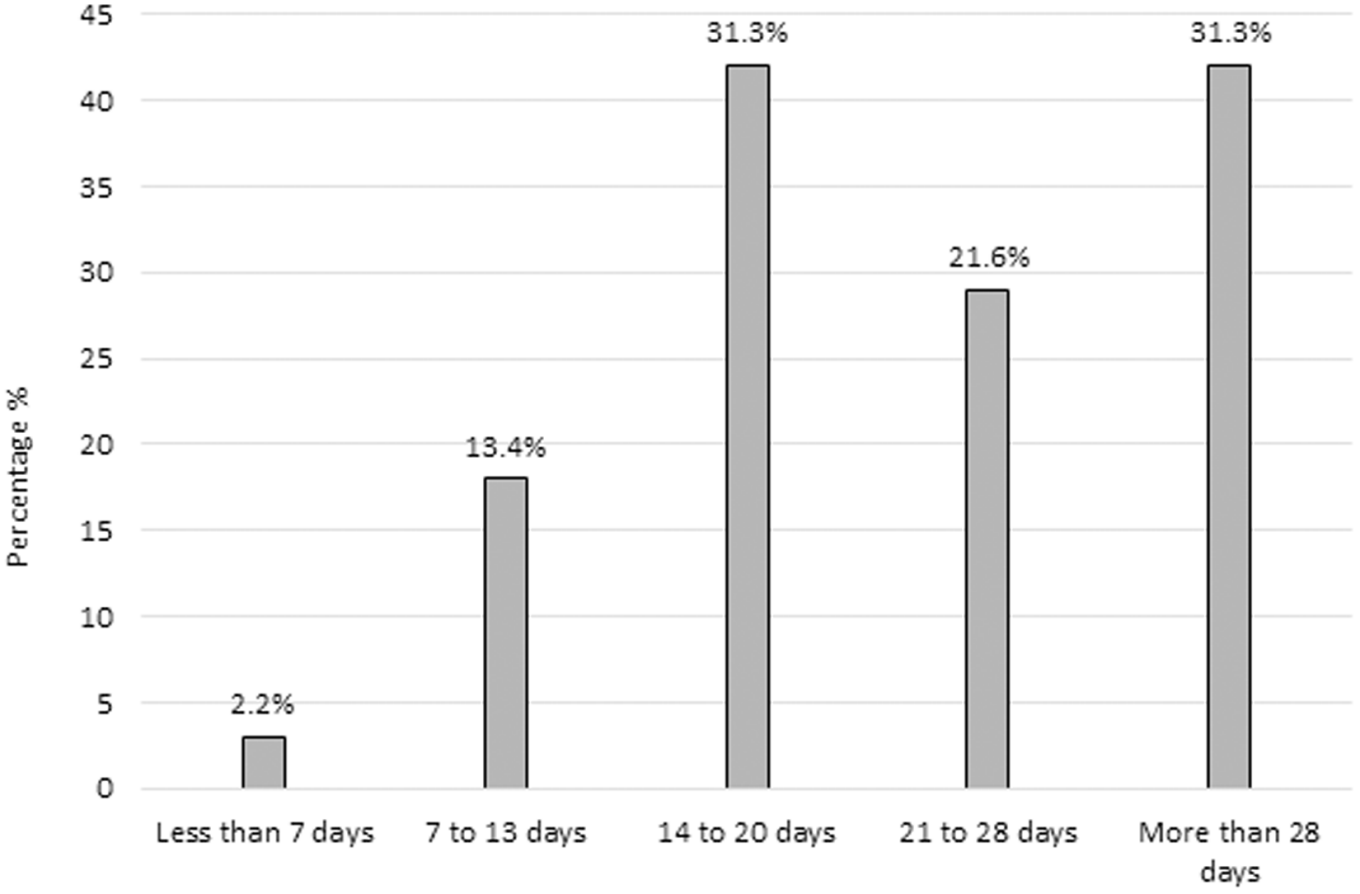
Time interval between injury and anterior cruciate ligament reconstruction among Peruvian orthopedic surgeons (n = 134).

We found that 50% (n = 67; 95% CI: 41.5–58.5) of respondents had more than 10 years of experience as an orthopedic surgeon, whereas 53.7% (n = 72; 95% CI: 45.2–62.1) had less than 5 years of experience in ACLR (Fig 2). Regarding the interval of time in which ACLR is performed from day of injury, 31.3% (n = 42; 95% CI: 24.0–39.8) of the participants reported preferring to perform surgery between day 14–20 and later than 28 days after injury. Only 2.2% (n = 3; 95% CI: 0.7–6.8) reported choosing to perform surgery within 7 days after injury (Fig 3).

**Fig 4:**
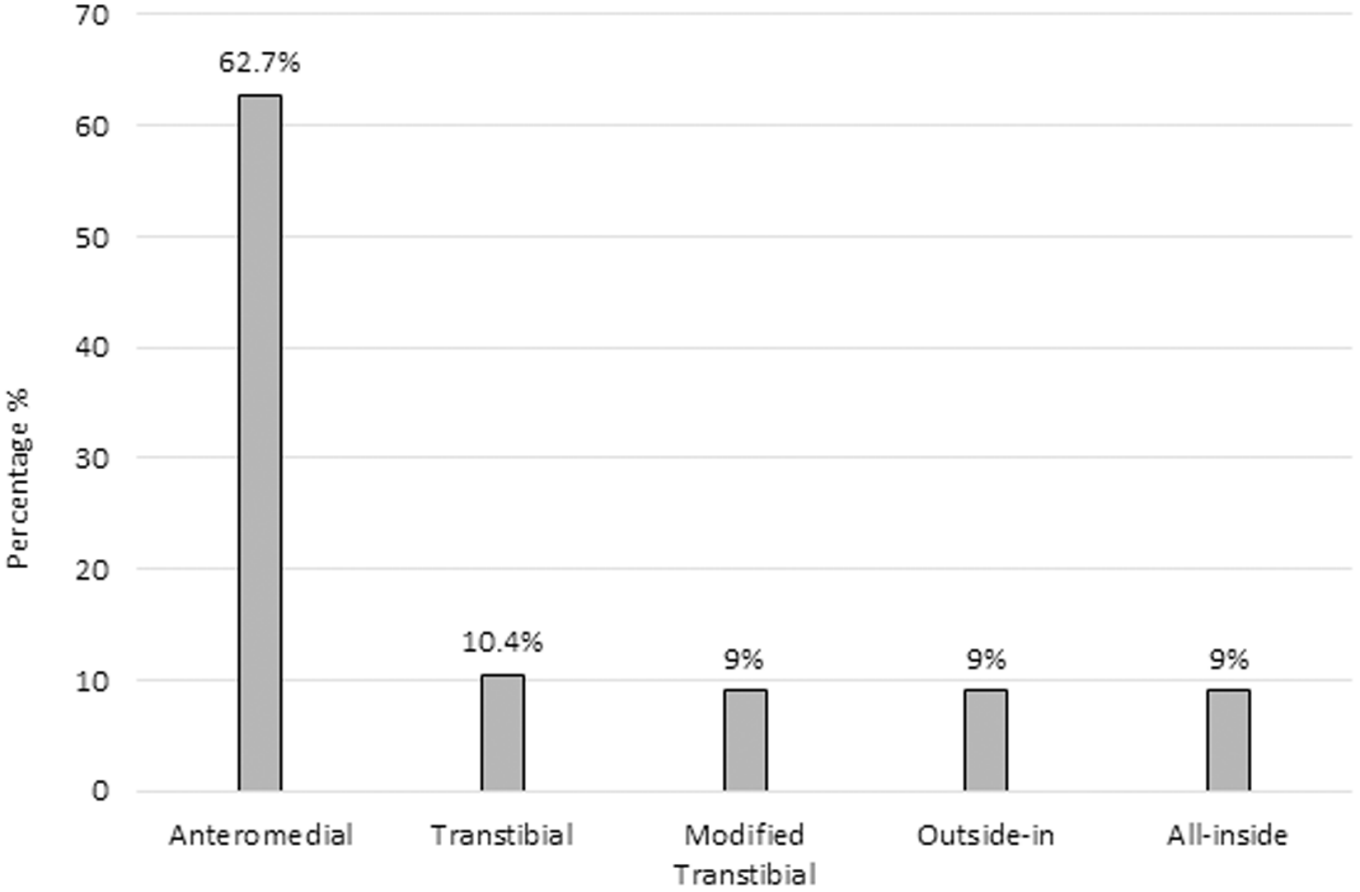
Preferred arthroscopic femoral tunnel drilling techniques in anterior cruciate ligament reconstruction (n = 134).

The position of femoral tunnel drilling using the anteromedial portal was the most preferred with 62.7% (n = 84; 95% CI: 54.1–70.5) of answers. In contrast, there were no significant differences between the other options of femoral tunnel drilling: TT, modified TT, OI, and AI (Fig 4). The use of autograft was the most common choice among the participants, representing 85.1% (n = 114; 95% CI: 77.9–90.2) of the total, while 13.4% (n = 18; 95% CI: 8.6–20.4) reported using allograft, and only 1.5% (n = 2; 95% CI: 0.4–5.8) preferred the use of a hybrid graft. Concerning the graft site of choice, 83.6% (n = 112; 95% CI: 76.2–89.0) reported preference for the hamstrings tendon, 16.4% (n = 22; 95% CI: 11.0–23.8) preferred using the patellar tendon, and none of the respondents reported using the quadriceps tendon or another type of graft.

**Fig 5:**
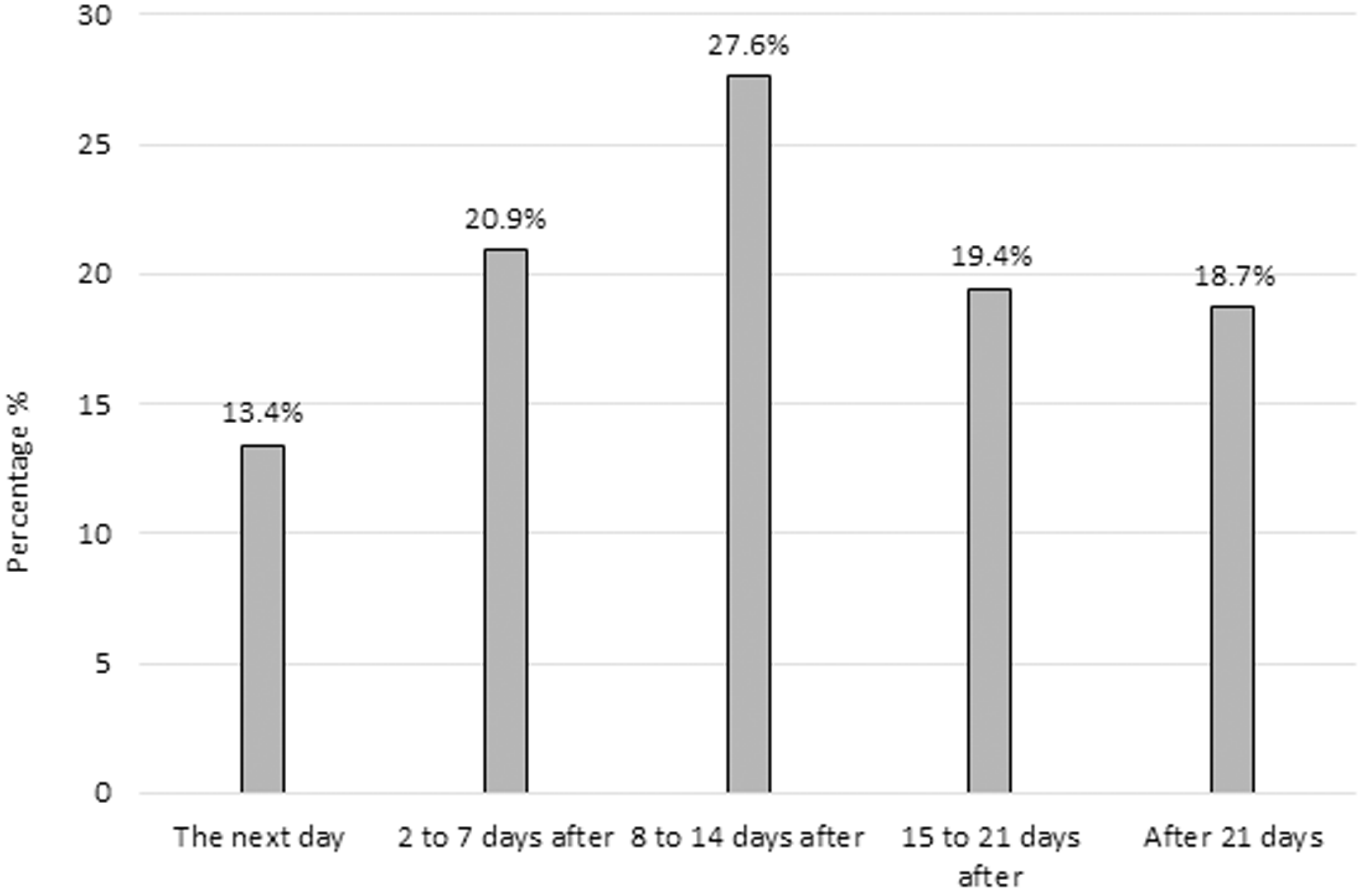
Time to initiate physical therapy following anterior cruciate ligament reconstruction (n = 134).

There were no distinct differences between the use of tourniquet. Thirty-four percent (n = 46; 95% CI: 26.7–42.8) reported never using a tourniquet during surgery, 37.3% (n = 50; 95% CI: 29.5–45.9) reported using it sometimes, and 28.4% (n = 38; 95% CI: 21.3–36.6) always used a tourniquet in their ACLR surgeries. It was found that 65.7% (n = 88; 95% CI: 57.2–73.3) of the surgeons surveyed did not use any compound after completion of the intervention. The most common compound used after surgery by 12.7% (n = 17; 95% CI: 8.0–19.5) of respondents was intra-articular corticosteroids, and 7.5% (n = 10; 95% CI: 4.0–13.4) of respondents reported using hyaluronic acid. Regarding the time interval before initiating physical therapy after surgery, 27.6% (n = 37; 95% CI: 20.7–35.9) of the respondents reported indicating physical therapy 8–14 days after surgery. The answers varied greatly with no clear differences between them (Fig 5).

## Discussion

This cross-sectional study identifies trends in ACL surgery in Peru and offers an initial exploratory overview of local surgical preferences. A total of 134 valid responses were collected. The majority of respondents were male (93.3%), and 47% were under 40 years of age, and over half (53%) worked in the private sector. Regarding experience, 50% had over 10 years of practice as orthopedic surgeons. In terms of surgical techniques, 62.7% preferred drilling the femoral tunnel using the anteromedial portal. The most common graft choice was autograft (85.1%), with the hamstring tendon being the most used (83.6%). Responses about tourniquet use were varied: 34% never used it, and 28.4% always did. Finally, 27.6% recommended starting physical therapy 8–14 days after surgery.

It is important to highlight that more half of the participants had more than 10 years of knee surgery expertise. Arthroscopic ACLR requires expertise and constant training. A lengthy learning curve with years of correctly practicing this procedure provides orthopedic surgeons sufficient experience to establish which technique and factors produce the most positive postoperative results [26,27]. In this survey 67 of the 134 responding surgeons had been orthopedic surgeons for more than 10 years, and among these surgeons, 35 had more than 10 years of experience with this particular procedure. One of the key steps in performing ACLR is the accuracy and precision of tibial and femoral tunnel placement, which may take more than 64 cases to achieve an adequate learning curve [28].

In the 1980s the gold standard procedure for tunnel placement during ACLR was transtibial (TT) drilling for making the tibial and femoral tunnel [16,29]. However, TT drilling was correlated with a rotational instability because the femoral tunnel position depends on the tibial tunnel orientation, creating a non-anatomic reconstruction of the ACL in some cases [16,29]. Studies have shown that the AM portal enables more anatomic femoral tunnel placement, improving rotational stability in many cases [16]. On comparing the TT vs. the AM technique, the latter showed to be significantly better with the IKDC subjective score, the Lachman test, and the pivot-shift sign [30,31]. Six out of ten members of the SPOT, who participated in this survey, preferred the AM technique. Nevertheless, one out of ten of the respondents still used the TT technique. The persistence of TT technique use among a subset of respondents may reflect variations in training, access to updated resources, or surgeon familiarity. Our study did not explore these factors in detail, highlighting a need for further research.

One of the most discussed choices in ACLR is the graft type. In our study, 83.6% of surgeons reported using hamstring tendon autografts, a higher proportion than that reported in similar international surveys [22]. This preference may reflect both surgeon familiarity and perceptions of reduced donor site morbidity [32]. Although bone–patellar tendon–bone grafts are associated with higher rates of anterior knee pain and extension loss [33], our study did not collect clinical outcome data to assess whether such complications influenced graft selection. Further local research could clarify the factors guiding these decisions.

One potentially unexpected finding was that 34% of respondents reported never using a tourniquet during ACL reconstruction. Intraoperative knee visualization, surgery time and postoperative pain could be some of the factors why an orthopedic surgeon chooses to use a tourniquet. In the present survey, 66% of the respondents always or sometimes used a tourniquet. Nevertheless, in a previous meta-analysis there were no significant differences between the use or not of a tourniquet for the intraoperative joint visualization [34]. Regarding the surgical time, Nakayama *et al.* reported no statistical difference in ACLR with or without the use of a torniquet [35]. Furthermore, immediate post-operative symptoms of pain were significantly higher at 4h and 10h following torniquet use according to the visual analog scale [36]. Further studies are needed to establish a strong recommendation of the intraoperative use of this flow restriction device.

Regarding the intraarticular compounds used after an ACLR, more than 65% of the participants perform this procedure without the use of any of the above-mentioned compounds. The use of these medications is to reduce postoperative pain, postoperative hemarthrosis or the range of motion [37]. It has been reported that the administration of an intra-articular corticosteroid after a traumatic event decreases the biomarkers of chondral degeneration with no increase in the risk of infection [38,39]. Nevertheless, the cytotoxic effects of corticosteroid administration is the principal concern for this therapy and should be widely evaluated before its use [40]. Eight of the respondents reported using tranexamic acid. A recent meta-analysis showed that in comparison to intraarticular administration of tranexamic acid only intravenous administration significantly reduced hemarthrosis and pain scores without increasing thromboembolic events [37]. The same number of participants reported using platelet-rich plasma (6%). According to these respondents, platelet-rich plasma was used to help the maturation of the graft in the bone. However, a systematic review showed that while the results were promising, a recommendation for its use could not yet be made [41]. Regarding the use of hyaluronic acid and bupivacaine for reducing postoperative pain, the use of the first showed a significant reduction during the first two weeks in comparison with a control group [42]. On the other hand, a meta-analysis reported that intra-articular administration of bupivacaine did not show a significant reduction in postoperative pain [43]. Nevertheless, bupivacaine in combination with morphine was found to reduce pain up to 12 hours after surgery with significantly lower postoperative opioid use [43]. Further studies comparing all these compounds are needed in order to make recommendations.

Currently, there is no strict recommendation on the type or time of initiation of physiotherapy [44]. However, postoperative physiotherapy is essential for patient recovery following ACLR and important for returning to sports activities [45]. Our results show that there is no concrete postoperative protocol, but eight out of ten of the surgeons interviewed prefer to start in the first three weeks after reconstruction, according to the current general recommendation of an early rehabilitation program [44].

This study presents the first comprehensive analysis of ACL reconstruction practices in Peru, revealing distinct patterns that differ from commonly reported practices in North American and European literature [46]. Notable findings include Peruvian surgeons’ preference for delayed surgical timing (14–20 days or beyond 28 days post-injury) rather than early intervention, and a strong predilection for hamstring autografts (83.6%) with a complete absence of quadriceps tendon usage, despite its growing international popularity [47]. These insights are particularly valuable as they highlight how surgical practices in a developing country may differ from more extensively studied regions, potentially reflecting variations in resource availability, training patterns, and clinical decision-making [46]. This baseline data about regional surgical preferences could help inform the development of more globally representative surgical guidelines and identify opportunities for educational interventions regarding newer techniques in ACL reconstruction.

The research results have potential applications which extend past academic purposes. National training programs and residency curricula should use this information to prioritize the techniques which surgeons most frequently use in Peru. The findings about graft selection and rehabilitation methods will help create standardized clinical guidelines which match the Peruvian healthcare environment. Future policy initiatives should concentrate on enhancing access to advanced graft solutions and unproven technologies that have not spread throughout our region.

While the results offer a snapshot of current practices, they also underscore the need for further research to standardize treatment protocols in ACLR. The variability in surgical timing, graft choices, and post-operative care (such as the use of compounds and physical therapy initiation) highlights areas where more evidence-based guidelines could be developed. Additionally, the differences in tourniquet use and the low rate of armed forces involvement suggest that broader studies may be needed to assess how these factors impact long-term outcomes. Future research should explore the benefits and disadvantages of different surgical approaches and graft sites, the role of post-operative treatments, and optimal rehabilitation timelines, to enhance the consistency and effectiveness of ACLR procedures across diverse clinical settings.

The main limitation of this study is the potential for selection bias. Although we attempted to reach all orthopedic surgeons performing ACL reconstruction within SPOT, the response rate was not high enough to be considered fully representative. This limited number of responses may affect the generalizability of the findings, as non-respondents could have different surgical practices or preferences compared to respondents. Although the survey did not cover the majority of SPOT members, there is a higher likelihood of finding specialists on this topic within this society at the Peruvian level. The results cannot be extrapolated to other countries because of the population type and accessibility of resources. For that reason, a comparison of all the points of the survey cannot be objectively made. This was an exploratory study and therefore our main interest was to identify ACLR preferences among a sample of Peruvian orthopedic surgeons. Consequently, no conclusions or suggestions for the most advantageous therapy can be inferred. Nevertheless, it should be noted that the ACL knee surgeons in Peru have the same trends as the other 57 countries reviewed [22]. Future studies could evaluate differences in preferences based on demographic and contextual factors, using diverse study designs to provide a more comprehensive understanding.

To our knowledge, this is the first national survey of ACLR practices in Peru, providing a valuable baseline on surgical preferences in the region. Notable findings include the high preference for hamstring autografts, frequent use of the anteromedial portal technique, and variability in the timing of physical therapy initiation, with most surgeons starting within the first three weeks. These trends contrast with some practices reported in high-income countries and may reflect contextual factors such as training, resource availability, and institutional protocols. Future research should investigate how these surgical preferences impact clinical outcomes, rehabilitation effectiveness, and long-term joint stability in the Peruvian population. Additionally, further studies could help inform the development of national guidelines and consensus statements tailored to regional needs.

## Supporting information

S1 File Survey. (DOCX)

## References

[1] Domnick C, Raschke MJ, Herbort M. Biomechanics of the anterior cruciate ligament: physiology, rupture and reconstruction techniques. World J Orthop. 2016;7(2):82–93. doi: 10.5312/wjo.v7.i2.82 26925379 PMC4757662

[2] Kaeding CC, Léger-St-Jean B, Magnussen RA. Epidemiology and diagnosis of anterior cruciate ligament injuries. Clin Sports Med. 2017;36(1):1–8. doi: 10.1016/j.csm.2016.08.001 27871652

[3] Leathers MP, Merz A, Wong J, Scott T, Wang JC, Hame SL. Trends and demographics in anterior cruciate ligament reconstruction in the United States. J Knee Surg. 2015;28(5):390–4. doi: 10.1055/s-0035-1544193 25635874

[4] Mall NA, Chalmers PN, Moric M, Tanaka MJ, Cole BJ, Bach Jr BR. Incidence and trends of anterior cruciate ligament reconstruction in the United States. Am J Sports Med. 2014;42:2363–70. 25086064 10.1177/0363546514542796

[5] Kiapour AM, Murray MM. Basic science of anterior cruciate ligament injury and repair. Bone Joint Res. 2014;3(2):20–31. doi: 10.1302/2046-3758.32.2000241 24497504 PMC3922117

[6] Kaeding CC, Aros B, Pedroza A, Pifel E, Amendola A, Andrish JT, et al. Allograft versus autograft anterior cruciate ligament reconstruction: predictors of failure from a MOON prospective longitudinal cohort. Sports Health. 2011;3(1):73–81. doi: 10.1177/1941738110386185 23015994 PMC3445196

[7] Maletis GB, Inacio MCS, Funahashi TT. Risk factors associated with revision and contralateral anterior cruciate ligament reconstructions in the Kaiser Permanente ACLR registry. Am J Sports Med. 2015;43(3):641–7. doi: 10.1177/0363546514561745 25548148

[8] Wang H-D, Gao S-J, Zhang Y-Z. Hamstring autograft versus hybrid graft for anterior cruciate ligament reconstruction: a systematic review. Am J Sports Med. 2020;48(4):1014–22. doi: 10.1177/0363546519849483 31166113

[9] Larson AI, Bullock DP, Pevny T. Comparison of 4 femoral tunnel drilling techniques in anterior cruciate ligament reconstruction. Arthroscopy. 2012;28(7):972–9. doi: 10.1016/j.arthro.2011.12.015 22409948

[10] Siegel L, Vandenakker-Albanese C, Siegel D. Anterior cruciate ligament injuries: anatomy, physiology, biomechanics, and management. Clin J Sport Med. 2012;22(4):349–55. doi: 10.1097/JSM.0b013e3182580cd0 22695402

[11] Tejwani SG, Prentice HA, Wyatt RWB Jr, Maletis GB. Femoral tunnel drilling method: risk of reoperation and revision after anterior cruciate ligament reconstruction. Am J Sports Med. 2018;46(14):3378–84. doi: 10.1177/0363546518805086 30419174

[12] Metso L, Nyrhinen K-M, Bister V, Sandelin J, Harilainen A. Comparison of clinical results of anteromedial and transtibial femoral tunnel drilling in ACL reconstruction. BMC Musculoskelet Disord. 2020;21(1):341. doi: 10.1186/s12891-020-03351-w 32493289 PMC7271541

[13] Alentorn-Geli E, Lajara F, Samitier G, Cugat R. The transtibial versus the anteromedial portal technique in the arthroscopic bone-patellar tendon-bone anterior cruciate ligament reconstruction. Knee Surg Sports Traumatol Arthrosc. 2010;18(8):1013–37. doi: 10.1007/s00167-009-0964-0 19902178

[14] Duquin TR, Wind WM, Fineberg MS, Smolinski RJ, Buyea CM. Current trends in anterior cruciate ligament reconstruction. J Knee Surg. 2009;22(1):7–12. doi: 10.1055/s-0030-1247719 19216345

[15] Bedi A, Musahl V, Steuber V, Kendoff D, Choi D, Allen AA, et al. Transtibial versus anteromedial portal reaming in anterior cruciate ligament reconstruction: an anatomic and biomechanical evaluation of surgical technique. Arthroscopy. 2011;27(3):380–90. doi: 10.1016/j.arthro.2010.07.018 21035990

[16] Vijayan S, Kyalakond H, Kulkarni MS, Aroor MN, Shetty S, Bhat V, et al. Clinical outcome of anterior cruciate ligament reconstruction with modified transtibial and anteromedial portal. Musculoskelet Surg. 2021:1–9. 10.1007/s12306-021-00727-6 PMC10020253 34389922

[17] Branam BR, Utz CJ. Indications for two-incision (outside-in) anterior cruciate ligament reconstruction. Clin Sports Med. 2017;36(1):71–86. doi: 10.1016/j.csm.2016.08.004 27871662

[18] Sim J-A, Kim J-M, Lee S, Bae J-Y, Seon J-K. Comparison of tunnel variability between trans-portal and outside-in techniques in ACL reconstruction. Knee Surg Sports Traumatol Arthrosc. 2017;25(4):1227–33. doi: 10.1007/s00167-015-3950-8 26713326

[19] Connaughton AJ, Geeslin AG, Uggen CW. All-inside ACL reconstruction: How does it compare to standard ACL reconstruction techniques? J Orthop. 2017;14(2):241–6. doi: 10.1016/j.jor.2017.03.002 28360487 PMC5360217

[20] Herzog MM, Marshall SW, Lund JL, Pate V, Mack CD, Spang JT. Trends in incidence of ACL reconstruction and concomitant procedures among commercially insured individuals in the United States, 2002-2014. Sports Health. 2018;10:523–31. 30355175 10.1177/1941738118803616 PMC6204641

[21] Musahl V, Becker R, Fu FH, Karlsson J. New trends in ACL research. KSSTA. 2011:1–3. 10.1007/s00167-011-1688-5 21946981

[22] Chechik O, Amar E, Khashan M, Lador R, Eyal G, Gold A. An international survey on anterior cruciate ligament reconstruction practices. Int Orthop. 2013;37(2):201–6. doi: 10.1007/s00264-012-1611-9 22782378 PMC3560905

[23] Grassi A, Carulli C, Innocenti M, Mosca M, Zaffagnini S, Bait C, et al. New trends in anterior cruciate ligament reconstruction: a systematic review of national surveys of the last 5 years. Joints. 2018;6(3):177–87. doi: 10.1055/s-0038-1672157 30582107 PMC6301855

[24] Martins LCG, Lopes MV de O, Diniz CM, Guedes NG. The factors related to a sedentary lifestyle: a meta-analysis review. J Adv Nurs. 2021;77(3):1188–205. doi: 10.1111/jan.14669 33368524

[25] Bengtsson H, Ortega Gallo PA, Ekstrand J. Injury epidemiology in professional football in South America compared with Europe. BMJ Open Sport Exerc Med. 2021;7(4):e001172. doi: 10.1136/bmjsem-2021-001172 34659791 PMC8488699

[26] Bhattacharyya R, Davidson DJ, Sugand K, Akhbari P, Bartlett MJ, Bhattacharya R, et al. Knee arthroscopy: a simulation demonstrating the Imperial Knee Arthroscopy Cognitive Task Analysis (IKACTA) Tool. JBJS Essent Surg Tech. 2018;8(4):e32. doi: 10.2106/JBJS.ST.18.00017 30775137 PMC6358334

[27] D’Ambrosi R, Carrozzo A, Meena A, Corona K, Yadav AK, Annibaldi A, et al. A slight degree of osteoarthritis appears to be present after anterior cruciate ligament reconstruction compared with contralateral healthy knees at a minimum of 20 years: A systematic review of the literature. J Exp Orthop. 2024;11: e12017. 10.1002/jeo2.12017 PMC10993150 38577065

[28] Luthringer TA, Blackmore SA, Singh BC, Strauss EJ. The learning curve associated with anteromedial portal drilling in ACL reconstruction. Phys Sportsmed. 2016;44(2):141–7. doi: 10.1080/00913847.2016.1154448 26882105

[29] Piasecki DP, Bach BR Jr, Espinoza Orias AA, Verma NN. Anterior cruciate ligament reconstruction: can anatomic femoral placement be achieved with a transtibial technique? Am J Sports Med. 2011;39(6):1306–15. doi: 10.1177/0363546510397170 21335345

[30] Alentorn-Geli E, Samitier G, Alvarez P, Steinbacher G, Cugat R. Anteromedial portal versus transtibial drilling techniques in ACL reconstruction: a blinded cross-sectional study at two- to five-year follow-up. Int Orthop. 2010;34(5):747–54. doi: 10.1007/s00264-010-1000-1 20401753 PMC2903180

[31] Lin KM, Boyle C, Marom N, Marx RG. Graft selection in anterior cruciate ligament reconstruction. Sports Med Arthrosc Rev. 2020;28(2):41–8. doi: 10.1097/JSA.0000000000000265 32345925

[32] Schoderbek RJ Jr, Treme GP, Miller MD. Bone-patella tendon-bone autograft anterior cruciate ligament reconstruction. Clin Sports Med. 2007;26(4):525–47. doi: 10.1016/j.csm.2007.06.006 17920951

[33] Mohtadi NGH, Chan DS, Dainty KN, Whelan DB. Patellar tendon versus hamstring tendon autograft for anterior cruciate ligament rupture in adults. Cochrane Database Syst Rev. 2011. 10.1002/14651858.CD005960.pub2 PMC6465162 21901700

[34] Wang J, Xu W, Lv J. Is it better to routinely use tourniquet for knee arthroscopic surgery: a systematic review and meta-analysis. J Knee Surg. 2020;33(9):866–74. doi: 10.1055/s-0039-1688555 31064020

[35] Nakayama H, Yoshiya S. The effect of tourniquet use on operative performance and early postoperative results of anatomic double-bundle anterior cruciate ligament reconstruction. J Orthop Sci. 2013;18(4):586–91. doi: 10.1007/s00776-013-0405-2 23686085

[36] Reda W, ElGuindy AMF, Zahry G, Faggal MS, Karim MA. Anterior cruciate ligament reconstruction; is a tourniquet necessary? A randomized controlled trial. KSSTA. 2016;24:2948–52. 10.1007/s00167-015-3582-z 25786826

[37] Johns WL, Walley KC, Hammoud S, Gonzalez TA, Ciccotti MG, Patel NK. Tranexamic acid in anterior cruciate ligament reconstruction: a systematic review and meta-analysis. Am J Sports Med. 2021;49(14):4030–41. doi: 10.1177/0363546521988943 33630652

[38] Lattermann C, Jacobs CA, Proffitt Bunnell M, Huston LJ, Gammon LG, Johnson DL, et al. A multicenter study of early anti-inflammatory treatment in patients with acute anterior cruciate ligament tear. Am J Sports Med. 2017;45(2):325–33. doi: 10.1177/0363546516666818 28146402

[39] Cantrell W, Swinehart S, Johnson C, Strnad G, Obuchowski N, Cox C. The impact of aspiration and corticosteroid injection after ACL injury on post-reconstruction infection rate. Orthop J Sports Med. 2021;9(161). 10.1177/03635465231211606 37975540

[40] Nakazawa F, Matsuno H, Yudoh K, Watanabe Y, Katayama R, Kimura T. Corticosteroid treatment induces chondrocyte apoptosis in an experimental arthritis model and in chondrocyte cultures. Clin Exp Rheumatol. 2002;20(6):773–81. 12508768

[41] Figueroa D, Figueroa F, Calvo R, Vaisman A, Ahumada X, Arellano S. Platelet-rich plasma use in anterior cruciate ligament surgery: systematic review of the literature. Arthroscopy. 2015;31:981–8. 25595696 10.1016/j.arthro.2014.11.022

[42] Chau JYM, Chan WL, Woo SB, Cheng SC, Wong TM, Wong TK, et al. Hyaluronic acid instillation following arthroscopic anterior cruciate ligament reconstruction: a double-blinded, randomised controlled study. J Orthop Surg (Hong Kong). 2012;20(2):162–5. doi: 10.1177/230949901202000205 22933671

[43] Davey MS, Hurley ET, Anil U, Moses A, Thompson K, Alaia M, et al. Pain management strategies after anterior cruciate ligament reconstruction: a systematic review with network meta-analysis. Arthroscopy. 2021;37(4):1290-1300.e6. doi: 10.1016/j.arthro.2021.01.023 33515736

[44] Badawy CR, Jan K, Beck EC, Fleet N, Taylor J, Ford K, et al. Contemporary principles for postoperative rehabilitation and return to sport for athletes undergoing anterior cruciate ligament reconstruction. Arthrosc Sports Med Rehabil. 2022;4(1):e103–13. doi: 10.1016/j.asmr.2021.11.002 35141542 PMC8811493

[45] Pereira M, Vieira N de S, Brandão E da R, Ruaro JA, Grignet RJ, Fréz AR. Physiotherapy after reconstruction of anterior cruciate ligament. Acta Ortop Bras. 2012;20(6):372–5. doi: 10.1590/S1413-78522012000600011 24453634 PMC3861958

[46] Bowman EN, Limpisvasti O, Cole BJ, ElAttrache NS. Anterior cruciate ligament reconstruction graft preference most dependent on patient age: a survey of United States surgeons. Arthroscopy. 2021;37(5):1559–66. doi: 10.1016/j.arthro.2021.01.042 33539983

[47] Tuca M, Valderrama I, Eriksson K, Tapasvi S. Current trends in anterior cruciate ligament surgery. A worldwide benchmark study. J ISAKOS. 2023;8(1):2–10. doi: 10.1016/j.jisako.2022.08.009 36154898

