# Supplementary material for: Surgical preferences in anterior cruciate ligament reconstruction: A cross-sectional study in a low-middle income country
Source: PLoS One. 2025 Jul 28;20(7):e0327966. doi: 10.1371/journal.pone.0327966 (PMC12303275; doi:10.1371/journal.pone.0327966)
Supplement: S1 File — (DOCX) [file pone.0327966.s001.docx]

**Survey in English**

**What is your gender?**

- Female
- Male

**What is your age?**

- Under 30 years
- 30 to 35 years
- 36 to 40 years
- 41 to 45 years
- 46 to 50 years
- 51 to 55 years
- 56 to 60 years
- 61 to 65 years
- 66 to 70 years
- Over 70 years

**In which department do you live?**
• Amazonas

• Áncash

• Apurímac

• Arequipa

• Ayacucho

• Cajamarca

• Callao

• Cusco

• Huancavelica

• Huánuco

• Ica

• Junín

• La Libertad

• Lambayeque

• Metropolitan Lima

• Provinces of Lima

• Loreto

• Madre de Dios

• Moquegua

• Pasco

• Piura

• Puno

• San Martín

• Tacna

• Tumbes

• Ucayali

**What sector do you work in?**
(If you work in more than one sector, select where you performed the most ACL reconstructions last year.)

- MINSA (Ministry of Health)
- EsSalud (Social Health Insurance)
- Private Clinic or Office
- Armed Forces

**How many years have you been a specialist in orthopedics and traumatology?**

- Less than 5 years
- Between 5 and 10 years
- Between 11 and 20 years
- More than 20 years

**How many years of experience, as a specialist, do you have in ACL reconstruction?**

- Less than 5 years
- Between 5 and 10 years
- Between 11 and 20 years
- More than 20 years

**What is the time interval in which you usually perform ACL reconstruction after the day of injury?**

- Less than 7 days
- Between 7 to 13 days
- Between 14 to 20 days
- Between 21 to 28 days
- More than 28 days

**What surgical technique do you prefer for ACL reconstruction?**

- Anteromedial portal
- Transtibial portal
- Modified transtibial portal
- Outside-in
- All-inside

**What type of graft do you most frequently use for ACL reconstruction?**

- Autograft
- Allograft
- Hybrid graft

**What type of graft do you most frequently use for ACL reconstruction?**

- Hamstring (semitendinosus-gracilis)
- Patellar tendon
- Quadriceps tendon with patellar bone block
- Quadriceps tendon without patellar bone block
- Other, specify: ___________________________

**Do you use a tourniquet during ACL reconstruction?**

- Always
- Sometimes
- Never

**Do you routinely use any of the following compounds at the end of ACL reconstruction?**
(You may select more than one option.)

- Intra-articular corticosteroid
- Tranexamic acid
- Platelet-rich plasma
- Hyaluronic acid
- Other, specify: ___________________________
- None

**How long after ACL reconstruction does your patient begin physical therapy?**

- The next day
- 2 to 7 days later
- 8 to 14 days later
- 15 to 21 days later
- After 21 days

**Encuesta en español**

**¿Cuál es su sexo?**

- Femenino
- Masculino

**¿Cuál es su edad?**

- Menos de 30 años
- 30 a 35 años
- 36 a 40 años
- 41 a 45 años
- 46 a 50 años
- 51 a 55 años
- 56 a 60 años
- 61 a 65 años
- 66 a 70 años
- Mayor de 70 años

**¿En qué departamento vive?**

- Amazonas
- Áncash
- Apurímac
- Arequipa
- Ayacucho
- Cajamarca
- Callao
- Cusco
- Huancavelica
- Huánuco
- Ica
- Junín
- La Libertad
- Lambayeque
- Lima Metropolitana
- Provincias de Lima
- Loreto
- Madre de Dios
- Moquegua
- Pasco
- Piura
- Puno
- San Martín
- Tacna
- Tumbes
- Ucayali

**¿En qué sector trabaja? (Si labora en más de un sector, seleccionar donde haya realizado la mayor cantidad de reconstrucciones de ligamento cruzado anterior en el último año)**

- MINSA (Ministerio de Salud)
- EsSalud (Seguro Social de Salud)
- Clínica o consultorio privado
- Fuerzas Armadas

**¿Cuántos años tiene como especialista en traumatología y ortopedia?**

- Menos de 5 años
- Entre 5 y 10 años
- Entre 11 y 20 años
- Más de 20 años

**¿Cuántos años de experiencia, como especialista, tiene en reconstrucción de LCA?**

- Menos de 5 años
- Entre 5 y 10 años
- Entre 11 y 20 años
- Más de 20 años

**¿Cuál es el intervalo de tiempo en el que normalmente realiza la reconstrucción de LCA después del día de la lesión?**

- Menos de 7 días
- Entre 7 y 13 días
- Entre 14 y 20 días
- Entre 21 y 28 días
- Más de 28 días

**¿Qué técnica quirúrgica prefiere para la reconstrucción de LCA?**

- Portal anteromedial
- Portal transtibial
- Portal transtibial modificado
- Outside-in (fuera dentro)
- All-inside (todo dentro)

**¿Qué tipo de injerto utiliza con mayor frecuencia para la reconstrucción de LCA?**

- Autoinjerto
- Aloinjerto
- Injerto híbrido

**¿Qué tipo de injerto utiliza con mayor frecuencia para la reconstrucción de LCA?**

- Tendones isquiotibiales (semitendinoso-gracilis)
- Tendón rotuliano
- Tendón del cuádriceps con bloque óseo rotuliano
- Tendón del cuádriceps sin bloque óseo rotuliano
- Otro, especifique: ___________________________

**¿Usa torniquete durante la reconstrucción de LCA?**

- Siempre
- A veces
- Nunca

**¿Utiliza de manera rutinaria alguno de los siguientes compuestos al finalizar la reconstrucción de LCA? (Puede seleccionar más de una opción)**

- Corticoide intraarticular
- Ácido tranexámico
- Plasma rico en plaquetas
- Ácido hialurónico
- Otro, especifique: ___________________________
- Ninguno

**¿Cuánto tiempo después de la reconstrucción de LCA inicia la fisioterapia su paciente?**

- Al día siguiente
- Entre 2 y 7 días después
- Entre 8 y 14 días después
- Entre 15 y 21 días después
- Después de 21 días
